# *In Vitro* and *In Vivo* Efficacies of the EGFR/MEK/ERK Signaling Inhibitors in the Treatment of Alveolar Echinococcosis

**DOI:** 10.1128/AAC.00341-20

**Published:** 2020-07-22

**Authors:** Zhe Cheng, Zhijian Xu, Huimin Tian, Fan Liu, Xiu Li, Damin Luo, Yanhai Wang

**Affiliations:** a State Key Laboratory of Cellular Stress Biology, School of Life Sciences, Xiamen University, Xiamen, Fujian, China; b Parasitology Research Laboratory, School of Life Sciences, Xiamen University, Xiamen, Fujian, China; c Medical College, Xiamen University, Xiamen, Fujian, China

**Keywords:** EGFR signaling, apoptosis, echinococcosis, germinative cells

## Abstract

Alveolar echinococcosis (AE), caused by the larval stage of the cestode Echinococcus multilocularis, is a lethal disease in humans. Novel therapeutic options are urgently needed since the current chemotherapy displays limited efficiency in AE treatment. In this study, we assessed the *in vitro* and *in vivo* effects of the epidermal growth factor receptor (EGFR)/MEK/extracellular signal-regulated kinase (ERK) signaling inhibitors, including BIBW2992, CI-1033, and U0126, on E. multilocularis. Our data showed that BIBW2992, CI-1033, and U0126 all displayed *in vitro* effects on the viability of the E. multilocularis metacestode. These inhibitors also showed protoscolicidal activities and caused severe ultrastructural alterations in the parasite. Moreover, BIBW2992 and CI-1033 exhibited potent proapoptotic effects on E. multilocularis metacestodes. Strikingly, a large portion of the apoptotic cells were found to be the germinative cells. *In vivo* studies showed that BIBW2992 and U0126 significantly reduced parasite burden, and the parasite obtained from BIBW2992-treated mice displayed impaired structural integrity of the germinal layer. In conclusion, these findings demonstrate the potential of EGFR-mediated signaling as a target for the development of novel anti-AE agents. The EGFR inhibitor BIBW2992 represents a promising drug candidate and/or a lead compound for anti-AE chemotherapy.

## Abstract (precis)

Alveolar echinococcosis (AE), caused by the larval stage of the cestode Echinococcus multilocularis, is a lethal disease in humans. Novel therapeutic options are urgently needed since the current chemotherapy displays limited efficiency in AE treatment. In this study, we assessed the *in vitro* and *in vivo* effects of the epidermal growth factor receptor (EGFR)/MEK/extracellular signal-regulated kinase (ERK) signaling inhibitors, including BIBW2992, CI-1033, and U0126, on E. multilocularis.

## INTRODUCTION

The disease alveolar echinococcosis (AE) is caused by infection with the metacestode larvae of the tapeworm Echinococcus multilocularis. Humans become infected by ingesting infective eggs produced by adult tapeworms. The subsequent tumor-like development of the metacestode is invasive and results in massive lesions mainly in liver and other host organs, including the lungs, kidneys, spleen, and brain. AE is considered to be the most lethal helminthiasis, and the mortality rate in humans apart from effective treatment is >90% within 10 years (1).

The preferred treatment strategy for AE is radical resection of the parasitic mass, which is always accompanied by chemotherapy. In inoperable cases, chemotherapy remains the only option. The only two anti-AE drugs licensed to date are the benzimidazole carbamate derivatives albendazole and mebendazole. However, benzimidazoles show limited efficiency against AE, and a long-term uptake of large doses of drugs is usually required to avoid the further growth and spread of metacestode tissue (2, 3). Thus, improved drug treatments are urgently needed.

Receptor tyrosine kinases (RTKs) are widely expressed transmembrane proteins that act as receptors for extracellular signaling molecules, e.g., growth factors. Upon ligand binding, RTKs undergo tyrosine phosphorylation and in turn initiate signal transduction through the activation of downstream components. RTK-mediated signaling pathways have critical functions in many vital cellular processes, including cell proliferation, survival, and migration (4).

In parasitic helminths, RTKs are believed to be essential for the parasite to exploit host-derived signal molecules for its own development, growth, and reproduction. Therefore, RTKs and relevant downstream cellular signaling components are considered promising druggable targets in anthelminthic chemotherapy (5–7). Furthermore, owing to their important roles in human carcinogenesis, much research has been conducted on the exploration of inhibitory compounds of RTK-mediated signaling pathways, leading to a wealth of inhibitors available for identifying potential lead anthelminthic drugs (8–11).

More recently, signaling pathways mediated by the insulin receptor, epidermal growth factor receptor (EGFR), and fibroblast growth factor receptor families were characterized in E. multilocularis and were suggested to play important roles in host-parasite interaction and in regulation of the larval development and growth of E. multilocularis. Some human RTK signaling inhibitors were reported to impair E. multilocularis signaling components and inhibited, at least *in vitro*, the growth of the parasite (12–14).

We have recently shown that the EGFR/extracellular signal-regulated kinase (ERK) signaling pathway promotes the proliferation of E. multilocularis germinative cells. The EGFR inhibitors BIBW2992 and CI-1033 and the MEK/ERK inhibitor U0126 were shown to target the parasite’s EGFR/ERK signaling and inhibit germinative cell proliferation in cultured metacestode larvae (13). Here, we assessed the *in vitro* parasiticidal efficacy of these compounds against E. multilocularis, and their *in vivo* chemotherapeutic effect was also evaluated in experimentally infected mice.

## RESULTS

### BIBW2992, CI-1033, and U0126 exhibit parasiticidal activity against *E. multilocularis* metacestodes and protoscoleces *in vitro*.

**FIG 1:**
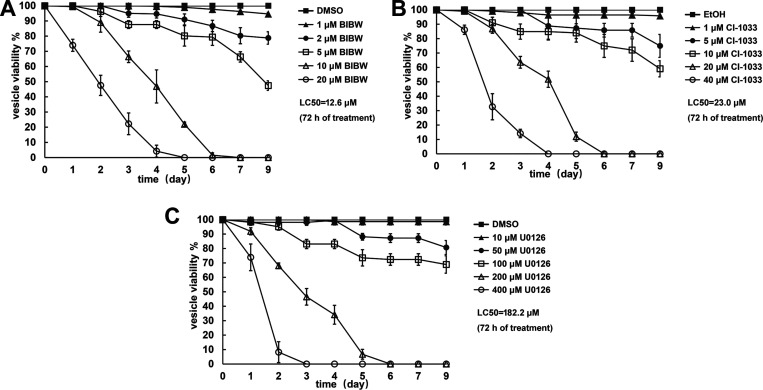
*In vitro* parasiticidal effect of BIBW2992, CI-1033, and U0126 on E. multilocularis metacestode vesicle. Viability of vesicles was assessed upon treatment with 1 to 20 μM BIBW2992 (A), 1 to 40 μM CI-1033 (B), or 10 to 400 μM U0126 (C) for the indicated times. The LC_50_ (after 72 h of treatment) of each drug is shown.

As shown in Fig. 1A and B, the EGFR inhibitors BIBW2992 and CI-1033 had a potent effect on the viability of *in vitro*-cultivated metacestode vesicles. The 50% lethal dose (LC_50_) values were 12.6 and 23.0 μM after 72 h of treatment with BIBW2992 and CI-1033, respectively. U0126 also exhibited a killing effect on metacestodes, whereas the LC_50_ value was relatively higher (182.2 μM) in comparison to those of the EGFR inhibitors (Fig. 1C). In addition, BIBW2992, CI-1033, and U0126 all showed *in vitro* activity against protoscoleces (see Fig. S1 in the supplemental material). Vesicles and protoscoleces treated with vehicle (control) showed little change in viability and morphology throughout the experimental period.

### BIBW2992, CI-1033, and U0126 altered the ultrastructure of *E. multilocularis* metacestodes.

**FIG 2:**
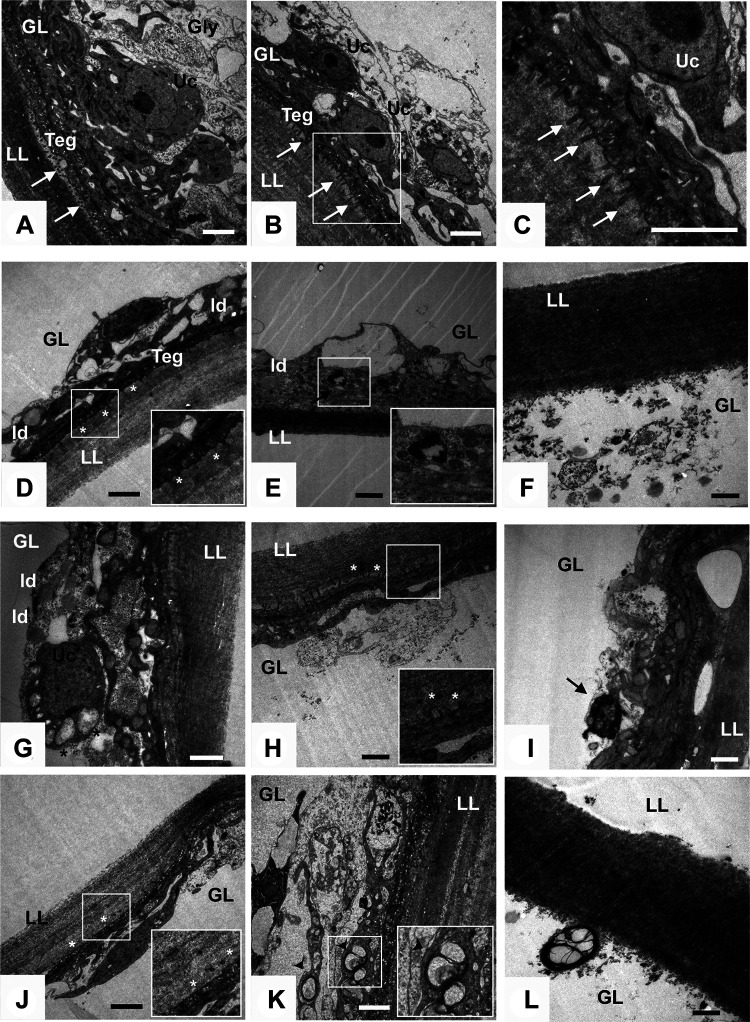
TEM images of E. multilocularis metacestode after *in vitro* treatment with BIBW2992, CI-1033, and U0126. Control vesicles treated with the solvent DMSO (A) or ethanol (EtOH) (B). (C) Enlarged view of the zone indicated in panel B. (D to F) Vesicles treated with BIBW2992 at concentrations of 0.5 μM for 72 h (D), 2 μM for 72 h (E), and 10 μM for 5 days (F). (G to I) Vesicles treated with 20 μM CI-1033 for 24 h (G), 48 h (H), and 72 h (I). (J to L) Vesicles treated with U0126 at concentrations of 100 μM for 24 h (J), 200 μM for 72 h (K), and 200 μM for 5 days (L). LL, laminated layer; GL, germinal layer; Teg, tegument; Uc, undifferentiated cells; Gly, glycogen storage cells; ld, lipid droplets. The white arrows in panels A to C indicate normal microtriches. White asterisks in panels D, H, and J indicate microtriches with a great reduction in number. Black arrows in panels E and I indicate cells with nuclear chromatin condensation and margination. The black asterisk in panel G indicates vacuolated cells. The black arrowheads in panel K indicate cells containing vacuoles and membrane stacks. Scale bar, 2 μm (for all figure panels).

The *in vitro* effects of the EGFR/ERK inhibitors were investigated by using transmission electron microscopy (TEM). No ultrastructural alteration was observed in control metacestode vesicles, which were surrounded by an acellular laminated layer, with numerous microtriches protruding from the tegument to the laminated layer (Fig. 2A to C). The interior layer (germinal layer) exhibited the typical appearance, which was constituted by a densely packed tissue containing connective tissue, undifferentiated cells (germinative cells) and glycogen storage cells. Distinct changes could be observed in the vesicles after 72 h of exposure to 0.5 μM BIBW2992, such as a less dense germinal layer tissue, a reduction in the number of the microtriches, and an increased number of lipid droplets (Fig. 2D). After 72 h of exposure to 2 μM BIBW2992, the majority of the microtriches were absent. Some cells were vacuolated or displayed nuclear chromatin condensation and margination (Fig. 2E), suggesting that cell death/apoptosis occurred in the parasite. Treatment with 10 μM BIBW2992 for 5 days resulted in complete destruction of the germinal layer tissue. Intact cells were hardly observed, and only the laminated layer and a few residues of tegumental tissue were left (Fig. 2F). Vesicles treated with CI-1033 exhibited similar ultrastructural alterations, including a less dense germinal layer, a reduced number of the microtriches, vacuolated cells, and cells with nuclear chromatin condensation and margination (Fig. 2G to I). U0126 treatment also showed dramatic effect, e.g., a greatly reduced number of the microtriches (100 μM for 24 h, Fig. 2J), an abnormal appearance of the germinal layer with cells containing vacuoles and membrane stacks (200 μM for 72 h, Fig. 2K), and the destruction of the germinal layer tissue (200 μM for 5 days, Fig. 2L).

### BIBW2992 and CI-1033 induced apoptosis in *E. multilocularis* metacestodes.

**FIG 3:**
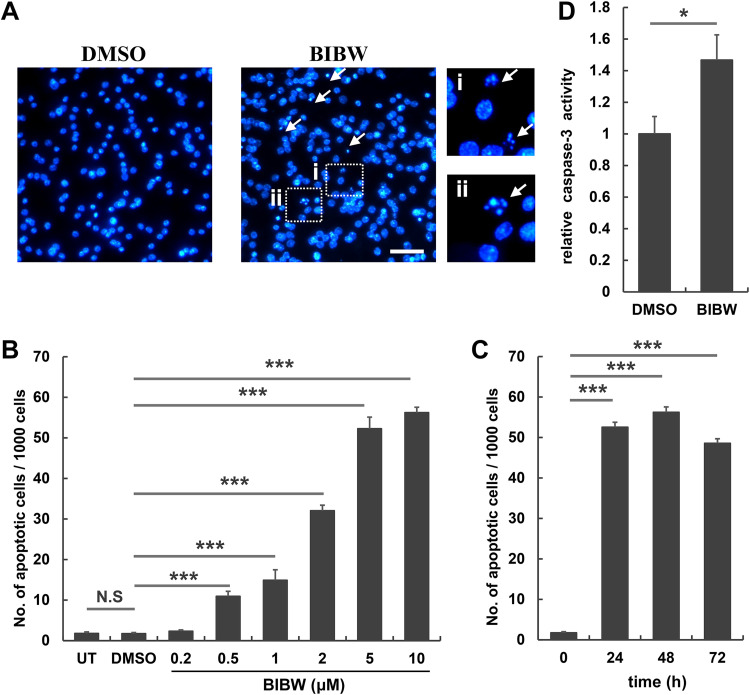
Assessment of proapoptotic effect of BIBW2992 on E. multilocularis metacestode vesicles *in vitro*. (A) DAPI staining images of vesicles treated with DMSO or 10 μM BIBW2992 (BIBW) for 48 h. Arrows indicate apoptotic cells. Scale bar, 20 μm. (B and C) Quantification of apoptotic cells in vesicles treated with 0.2 to 10 μM BIBW2992 for 48 h (B) or 10 μM BIBW2992 for 24 to 72 h (C). UT, untreated. (D) Caspase-3 activity of vesicles after exposure to 10 μM BIBW2992 for 48 h. Data in panels B to D are shown as means ± the standard deviations (SD). *, *P* < 0.05; ***, *P* < 0.001; N.S, not significant.

TEM revealed ultrastructural changes indicative of apoptosis in E. multilocularis metacestodes upon the treatment with BIBW2992 and CI-1033. We then analyzed the apoptotic activity of these drugs in the parasite. DAPI (4′,6′-diamidino-2-phenylindole) staining showed that BIBW2992-treated vesicles exhibited an increased number of apoptotic cells with condensed, fragmented nuclei and/or apoptotic bodies (Fig. 3A). Only a few apoptotic cells were detected in untreated or dimethyl sulfoxide (DMSO)-treated vesicles (1.7‰ ± 0.3‰ of total cells), whereas a significantly increased number of apoptotic cells could be observed after 48 h of exposure to 0.5 μM BIBW2992. BIBW2992 induced apoptosis in a dose-dependent manner, and the most prominent proapoptotic effect was observed upon treatment with 10 μM BIBW2992 for 48 h (56.2‰ ± 1.3‰ of total cells) (Fig. 3B and C). The activity of caspase-3 was also significantly increased in the vesicles treated with BIBW2992 (Fig. 3D). In addition, CI-1033 displayed a proapoptotic activity similar to that of BIBW2992 (Fig. S2A and B). These results indicate that both BIBW2992 and CI-1033 can induce apoptosis in E. multilocularis metacestodes.

Interestingly, we found no significantly increased apoptosis in the vesicles treated with 20 to 200 μM U0126 over 72 h of treatment.

### BIBW2992 and CI-1033 induced germinative cell apoptosis in *E. multilocularis*.

**FIG 4:**
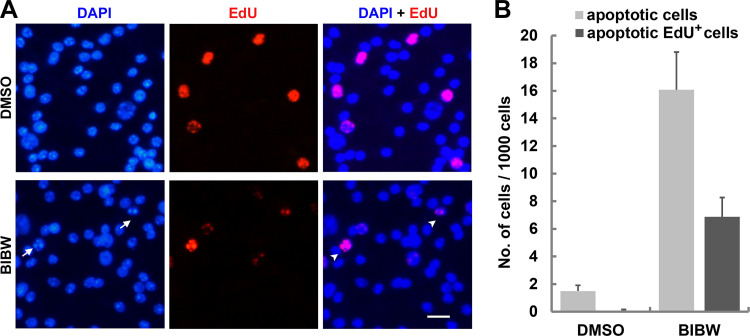
Proapoptotic effect of BIBW2992 on E. multilocularis germinative cells. (A) Vesicles were administered to a 4-h EdU pulse to label proliferating germinative cells (red) and then treated with DMSO or 10 μM BIBW2992 for another 8 h. Arrows indicate apoptotic EdU^+^ cells. Scale bar, 10 μm. (B) Quantification of apoptotic EdU^+^ cells in DMSO- or BIBW2992-treated vesicles. The data are shown as means ± the SD.

A population of adult stem cells, the germinative cells, are the only proliferative cells in E. multilocularis and are considered to drive growth and development of the parasite within the hosts (15). To investigate whether BIBW2992 induces germinative cell apoptosis, metacestode vesicles were exposed to a 4-h pulse of EdU (5-ethynyl-2′-deoxyuridine), an analogue of thymidine, to label the proliferating germinative cells and were then treated with BIBW2992 for another 8 h. The results showed that 16.1‰ ± 2.7‰ of the total cells underwent apoptosis after exposure to BIBW2992. Among these apoptotic cells, 42.5% ± 7.4% were EdU^+^. The apoptotic EdU^+^ cells were rarely presented in DMSO-treated vesicles (Fig. 4). Considering that the EdU^+^ cells were 6.2% of the total cells in the assay, these results suggest that the germinative cells were more sensitive to BIBW2992-induced apoptotic effect compared to differentiated cells. We also performed *in situ* TUNEL (terminal deoxynucleotidyltransferase-mediated dUTP-biotin nick end labeling) assay and observed the EdU^+^ cells that were TUNEL positive (Fig. S3). In addition, we also observed apoptotic EdU^+^ cells in CI-1033-treated vesicles (Fig. S2C). These results demonstrate that BIBW2992 and CI-1033 can induce germinative cell apoptosis in E. multilocularis.

### BIBW2992 and U0126 showed *in vivo* activity against *E. multilocularis*.

**FIG 5:**
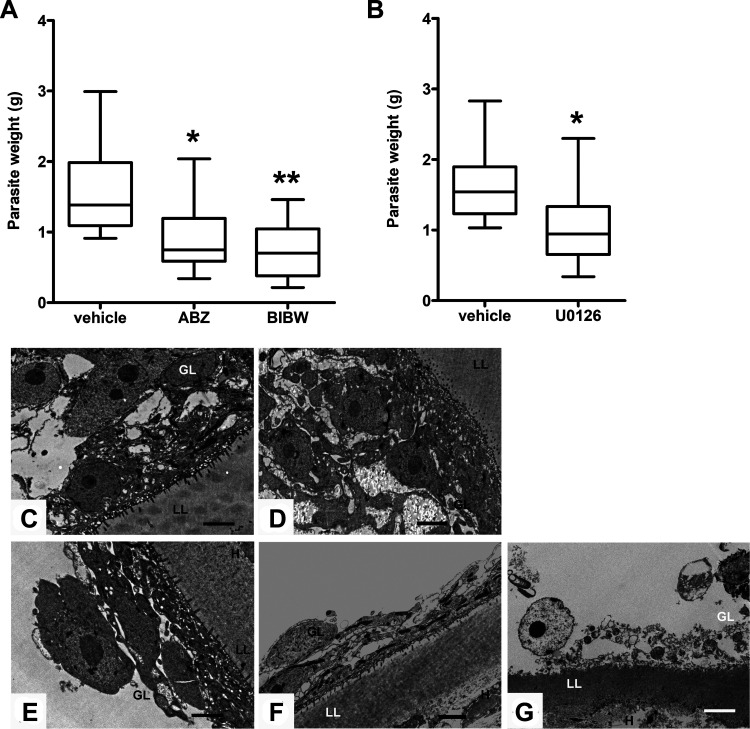
*In vivo* efficacies of BIBW2992 and U0126 against E. multilocularis in experimentally infected BALB/c mice. All treatments were performed 8 weeks postinfection and proceeded for 6 weeks. Each treatment group comprised 10 mice. (A) Mice were orally given 0.5% MC plus 0.5% CMC (vehicle control), albendazole (ABZ) in CMC (200 mg/kg/day), or BIBW2992 in MC (25 mg/kg/day 5 days a week). (B) Mice were given intraperitoneally 40% DMSO (vehicle control) or U0126 (25 μmol/kg three times per week). After euthanasia, parasite tissue was resected and weighed. Parasite weights are shown as box plots. *, *P* < 0.05; **, *P* < 0.01. (C to G) TEM images of E. multilocularis metacestodes obtained from vehicle-treated mice (C, MC + CMC; D, DMSO), albendazole-treated mice (E), U0126-treated mice (F), and BIBW2992-treated mice (G). LL, laminated layer; GL, germinal layer; H, host tissue. Scale bar, 2 μm.

The *in vivo* activity of BIBW2992 and U0126 against the E. multilocularis metacestode was investigated in BALB/c mice. Albendazole, as expected, significantly reduced parasite weights (42.7% mean reduction, *P* = 0.021 [compared to the vehicle control]). BIBW2992 exhibited a higher efficiency in reducing the parasite burden (53.5% mean reduction, *P* = 0.003 [compared to the vehicle control]) (Fig. 5A). Intraperitoneal administration of U0126 also resulted in a significantly reduced parasite weight (*P* = 0.032); however, the mean reduction (34.9%) was lower compared to BIBW2992 treatment (Fig. 5B).

The *in vivo* activities of albendazole, BIBW2992, and U0126 were also investigated by TEM. In comparison to vehicle-treated mice (Fig. 5C and D), parasites obtained from albendazole- and U0126-treated mice showed a thinner germinal layer and a reduced number of microtriches (Fig. 5E and F). BIBW2992 treatment exhibited even more dramatic effects on the parasite. Most of the microtriches were absent. A large part of the germinal tissue was not structurally intact, with only cellular debris remaining, suggesting that the viability of parasite cysts was impaired (Fig. 5G).

## DISCUSSION

Receptor tyrosine kinase signaling pathways have been suggested as promising targets in anthelminthic chemotherapy. Numerous inhibitors of human RTK signaling are currently available, and some of them are being pursued as anthelminthic agents through repurposing (7, 8, 10, 11).

While a few studies have been conducted on EGFR-targeted compounds against parasitic helminths (8, 16), we recently demonstrated that CI-1033, BIBW2992, and U0126 can inhibit the signaling activity of EGFR/MEK/ERK in E. multilocularis (13). We show here that these drugs exhibited parasiticidal activities against E. multilocularis metacestode vesicles and protoscoleces (PSCs) *in vitro* (Fig. 1; see also Fig. S1 in the supplemental material).

BIBW2992, also known as afatinib, is a potent EGFR inhibitor approved for the treatment of human non-small-cell lung cancer. We have previously shown that the growth of *in vitro*-cultivated E. multilocularis metacestode could be significantly inhibited by BIBW2992 at the concentrations of 0.1 to 0.5 μM (13), which are close to the maximum achievable drug plasma concentration (300 nM) (17). In the present study, the LC_50_ values of BIBW2992 were 12.6 and 9.3 μM against the metacestode vesicle and PSC, respectively. Distinct ultrastructural alterations could be observed upon treatment with lower concentrations, e.g., 0.5 μM for 72 h (Fig. 2). Moreover, oral application of BIBW2992 resulted in significantly reduced parasite weight in experimentally infected mice, with the dosage consistent with those used to treat malignant tumors (18–20). Although the difference of reduction in parasite burden was not statistically significant between BIBW2992- and albendazole-treated mice, TEM results showed that BIBW2992-treated parasite exhibited much more severe ultrastructural alterations, suggestive of the impaired viability of parasite cysts in mice (Fig. 5). In addition, CI-1033 displayed very similar *in vitro* effects as BIBW2992. Together, these results present E. multilocularis EGFR as a druggable target and suggest BIBW2992 and CI-1033, especially BIBW2992, as promising novel anti-AE agents and/or as lead compounds for AE treatment.

EGFR-mediated activation of the Ras/Raf/MEK/ERK signaling pathway has been demonstrated in E. multilocularis, and some drugs have been shown to inhibit the activation of the signaling in the parasite (13, 21, 22). U0126 was recently shown to display significant *in vitro* effect on protoscoleces of the closely related species *E. granulosus* and reduce, but not significantly, parasite burden in mice (23). In the present study, U0126 treatment resulted in significantly reduced parasite weight, despite the lower reduction in parasite burden in comparison with BIBW2992 and albendazole treatments. Several factors may cause this inconsistency. First, the ways of drug administration are different (intraperitoneal in this study versus peroral in theirs). Second, the treatment regimens are different (treatment was performed at 8 weeks postinfection in this study versus 6 months in theirs). Third, the developmental characteristics of the larvae of the two species are different. Low concentrations of U0126 (e.g., 20 to 40 μM) were previously shown to effectively suppress ERK phosphorylation and germinative cell proliferation in E. multilocularis and significantly inhibited the growth of cultured metacestode vesicles (13). In the present study, parasite cysts obtained from U0126-treated mice exhibited several ultrastructural alterations. Although these results suggest MEK/ERK as pharmacologically validated drug targets against echinococcosis, considering the relatively high LC_50_ values of U0126 presented in the *in vitro* studies and its modest *in vivo* effects, further improvements, such as improving its solubility and developing new formulations, are needed for the clinical use of U0126, and more efficient inhibitors against *Echinococcus* MEK/ERK signaling need to be identified and developed.

Strikingly, we found that BIBW2992 and CI-1033 displayed potent proapoptotic activities in E. multilocularis metacestode *in vitro* (Fig. 3; see Fig. S2 in the supplemental material). We could also observe cells that displayed apoptotic phenotypes in the parasite tissue obtained from BIBW2992-treated mice by TEM. These results offer a cellular mechanism of the drugs against E. multilocularis. The germinative cells are decisive for the tumor-like growth of E. multilocularis larvae within the host organs. Since benzimidazoles have limited effects on killing germinative cells, these cells have therefore emerged as a crucial target for the development of novel chemotherapeutics against AE (15, 24). BIBW2992 and CI-1033 have been shown to inhibit the proliferation of E. multilocularis germinative cells (13). We show here that these drugs exerted proapoptotic effects on germinative cells (Fig. 4; see Fig. S2 in the supplemental material), suggesting that they can also impair the survival and maintenance of germinative cells and may in turn inhibit tissue turnover and survival of E. multilocularis larvae in the host.

High levels of apoptosis have been suggested to be correlated with infertility of *E. granulosus* hydatid cysts (25, 26). A few drugs, e.g., dexamethasone, albendazole sulfoxide, and nanolipid carrier-loaded ivermectin, were shown to induce apoptosis in *E. granulosus* (27–29). Inducing apoptosis in the parasite may therefore provide a novel therapeutic strategy against echinococcosis. However, cellular components and mechanisms regulating apoptosis in *Echinococcus* spp. are largely unknown. In this study, our data define the EGFR signaling as a promising drug target for inducing apoptosis in E. multilocularis. Interestingly, we found no significantly increased apoptosis in *in vitro*-cultivated vesicles upon U0126 treatment. In mammals, downstream of EGFR lie the MEK/ERK, PI3K (phosphatidylinositol 3-kinase)/AKT, and STAT3 signaling pathways. The PI3K/AKT signaling has been recently identified in E. multilocularis (12), while the major components of STAT signaling have been shown to be absent in *Echinococcus* (and other cestodes) (30). It is very tempting to investigate the proapoptotic effect of drugs targeting the PI3K/AKT signaling pathway in E. multilocularis in future.

In summary, we demonstrate here the *in vitro* and *in vivo* activities of the EGFR/MEK/ERK signaling inhibitors BIBW2992, CI-1033, and U0126 against E. multilocularis. In particular, BIBW2992 may be considered as a novel drug candidate for AE treatment and/or as a lead compound for the development of EGFR-targeted drugs for AE chemotherapy.

## MATERIALS AND METHODS

### Ethics.

All animal experiments were performed strictly in accordance with China regulations on the protection of experimental animals and specifically approved by the Institutional Animal Care and Use Committee of Xiamen University (permit 2013-0053).

### *In vitro* culture of *E. multilocularis* and drug treatment.

*In vitro* cultivation of metacestode vesicles and protoscoleces was performed using host cell conditioned medium according to a previously established method (31). For drug treatment, viable vesicles (diameter, ca. 2 to 3 mm) and protoscoleces (PSCs) were cultured in conditioned medium containing BIBW2992 (1, 2, 5, 10, and 20 μM), CI-1033 (1, 5, 10, 20, and 40 μM), or U0126 (10, 50, 100, 200, and 400 μM). All drugs were supplied by Selleck Chemicals. Medium containing only DMSO (solvent of BIBW2992 and U0126; final concentration, 0.2%) or ethanol (solvent of CI-1033; final concentration, 0.1%) was used as the vehicle control. All experiments were performed with exchange of the medium containing the same ingredients every 2 days. The viability of vesicles was assessed on the basis of structural vesicle integrity, as previously described (32). The viability of PSCs was assessed by motile behavior observation and 0.1% methylene blue staining (33). More than 50 vesicles and 200 PSCs for each treatment group were analyzed, and three independent experiments were performed. The results were analyzed by a nonlinear curve fit method, and the 50% inhibitory concentrations were calculated using GraphPad Prism software.

### Transmission electron microscopy.

After exposure to drugs, metacestode vesicles were processed for TEM analysis as previously described with modifications (34). Briefly, vesicles were fixed for 2 h in 2.5% glutaraldehyde in 0.1 M phosphate-buffered saline (PBS [pH 7.4]) at room temperature and postfixed for 2 h in 1% OsO_4_ in 0.1 M PBS. Samples were washed with distilled water, dehydrated in an ethanol gradient, and then embedded in Epon 812 Spurr resin. Ultrathin sections were collected on a copper grid, stained with uranyl acetate and lead citrate, and examined by a HT-7800 transmission electron microscope (Hitachi).

### Apoptosis assay and EdU labeling.

Vesicles were incubated in medium supplemented with BIBW2992 (0.2 to 10 μM), CI-1033 (0.5 to 20 μM), U0126 (20 to 200 μM), or solvent for 24 to 72 h. Samples were then whole-mount prepared as previously described (13) and stained with 1 μg/ml DAPI for 15 min at room temperature to monitor apoptosis-associated chromatin condensation and apoptotic bodies. Then, four to seven random microscopic fields of one vesicle were imaged, and the numbers of apoptotic cells and total nuclei were counted. Six vesicles and >10,000 nuclei in total were analyzed for each treatment group, and three separate experiments were performed.

Caspase activity assay was conducted using the caspase-3 activity colorimetric assay kit (Beyotime Biotechnology, Shanghai, China). Briefly, vesicles were treated with 10 μM BIBW2992 or DMSO for 48 h. After three washes with PBS, lysates of vesicles were produced in lysis buffer, kept on ice for 20 min, and then centrifuged at 4°C and 17,000 × *g* for 10 min. The supernatants were used for caspase-3 activity assay according to the kit instructions.

EdU labeling was performed as previously described (13). Briefly, vesicles were incubated with 50 μM EdU for 4 h, washed thoroughly, and then treated with 10 μM BIBW2992 or DMSO for another 8 h. Samples were then fixed and whole mount prepared for the detection of EdU. A TUNEL assay was performed before EdU detection, as previously described (35). DNA was counterstained with DAPI. To quantify apoptotic EdU^+^ cells, the numbers of apoptotic cells and EdU^+^ and DAPI^+^ nuclei were determined from four to seven random microscopic fields of one vesicle. Six vesicles for each control, and treatment group were analyzed and two independent labeling experiments were performed.

### Mouse infection and *in vivo* treatment.

Experimental infection of mice with E. multilocularis was carried out by intraperitoneal injection of protoscoleces, as described previously (16). Briefly, PSCs were collected from parasite tissue. Each mouse (8-week-old female BALB/c mice) was infected intraperitoneally with ∼1,500 viable PSCs suspended in 150 μl of PBS containing 100 U/ml of penicillin and 100 μg/ml streptomycin. After 8 weeks, the mice were randomly divided into five groups of ten mice each. Groups of mice received their respective treatments: BIBW2992 (25 mg/kg/day 5 days a week, in 0.5% methylcellulose [MC], orally) (18), albendazole (200 mg/kg/day, in 0.5% carboxymethyl cellulose [CMC], orally) (36), 0.5% MC plus 0.5% CMC (vehicle control for BIBW2992 and albendazole, orally), U0126 (25 μmol/kg three times per week, in 40% DMSO, intraperitoneally) (37), and 40% DMSO (vehicle control for U0126, intraperitoneally). All treatments were performed for 6 weeks. At the end of the treatment, the mice were euthanized. Necroscopy was performed immediately, and the total parasite material was collected to measure the parasite weight. Some parasite cysts of each group were processed for TEM analysis.

### Statistics.

Experimental data were analyzed, and a two-tailed Student *t* test was used to determine the statistical significance between the control group and each treatment group. *P* values are indicated by asterisks in the figures (*, *P* < 0.05; **, *P* < 0.01, and ***, *P* < 0.001).

## Supplementary Material

Supplemental file 1
