# Supplementary material for: In Vitro and In Vivo Efficacies of the EGFR/MEK/ERK Signaling Inhibitors in the Treatment of Alveolar Echinococcosis
Source: Antimicrob Agents Chemother. 2020 Jul 22;64(8):e00341-20. doi: 10.1128/AAC.00341-20 (PMC7526812; doi:10.1128/AAC.00341-20)
Supplement: Supplemental file 1 [file AAC.00341-20-s0001.pdf]

Supplemental Material

Supplementary Figure S1-S3

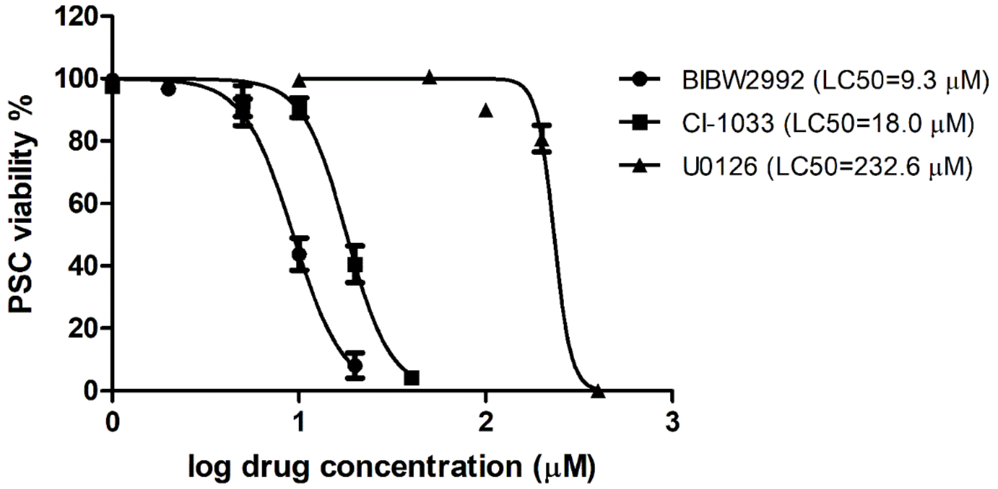

Figure S1 ***In vitro*** **parasiticidal effect of BIBW2992, CI-1033 and U0126 on** ***E. multilocularis*** **protoscoleces.**

Protoscoleces were treated with BIBW2992 (1, 2, 5, 10 and 20 μM), CI-1033 (1, 5, 10, 20, and 40 μM) or U0126 (10, 50, 100, 200 and 400 μM) and viability of protoscoleces was assessed after 72 h of treatment. Results were analyzed by nonlinear curve fit method and shown as log concentration–response curve. LC_{50} of each drug was determined and indicated in the figure.

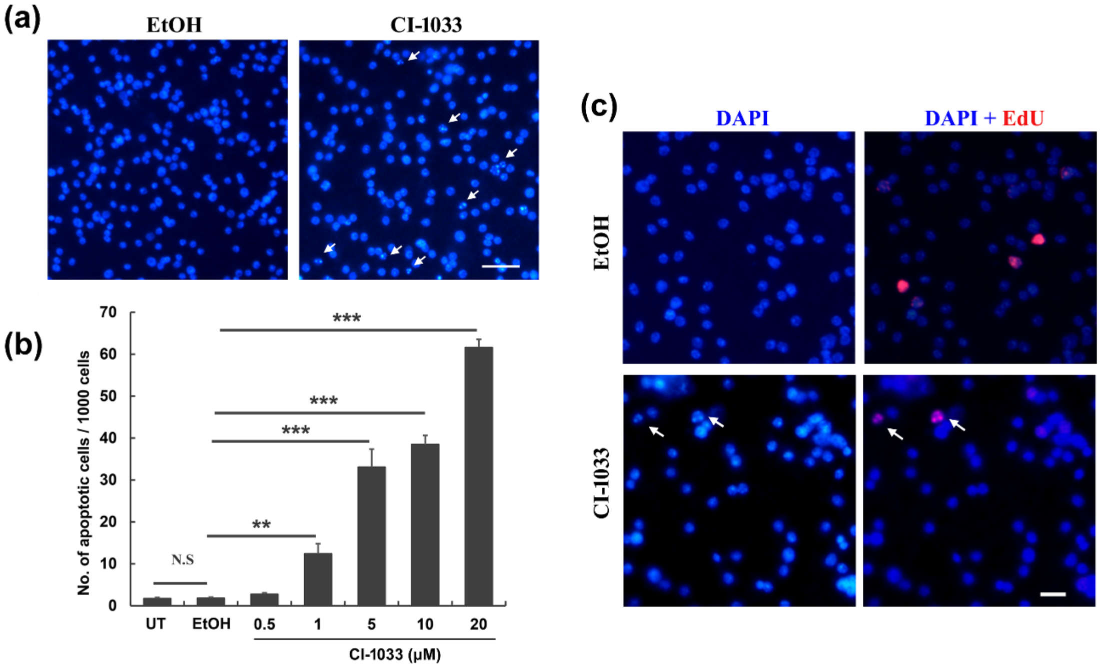

Figure S2 Assessment of proapoptotic effect of CI-1033 on *E. multilocularis* metacestode vesicles *in vitro*.

(A) DAPI staining images of vesicles treated with EtOH or 20 μM of CI-1033 for 24 h. Arrows indicate apoptotic cells. Bar=20 μm.

(B) Quantification of apoptotic cells in vesicles treated with EtOH or 0.5-20 μM of CI-1033 for 48 h. UT, untreated. Data are shown as mean ± SD. ** $P < 0.01$. *** $P < 0.001$. N.S, not significant.

(C) CI-1033 induced germinative cell apoptosis. Vesicles were administrated to a 4-h EdU pulse to label proliferating germinative cells (red), followed by exposure to EtOH or 20 μM of CI-1033 for another 8 h. Arrows indicate apoptotic EdU^{+} cells. Bar=10 μm.

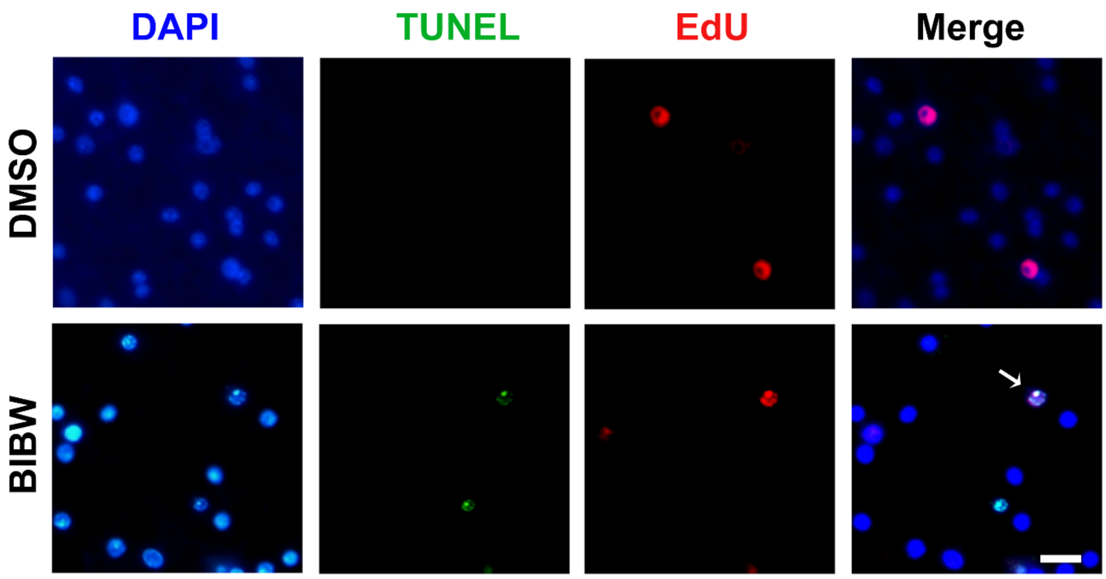

Figure S3 TUNEL assay of proapoptotic effect of BIBW2992 on *E. multilocularis* germinative cells.

Vesicles were administrated to a 4-h EdU pulse to label proliferating germinative cells and then treated with DMSO or 10 µM of BIBW2992 for another 8 h. Arrow indicates a TUNEL positive (green) cell that was also EdU^+ (red). Bar=10 µm.
